# Retraction Note: Performance of graphene-zinc oxide nanocomposite coated-glassy carbon electrode in the sensitive determination of para-nitrophenol

**DOI:** 10.1038/s41598-024-73187-0

**Published:** 2024-09-24

**Authors:** Riyaz Ahmad Dar, Gowhar Ahmad Naikoo, Ashwini Kumar Srivastava, Israr Ul Hassan, Shashi P. Karna, Lily Giri, Ahamad M. H. Shaikh, Mashallah Rezakazemi, Waqar Ahmed

**Affiliations:** 1 Department of Chemistry, Maharashtra College of Arts, Science and Commerce, Mumbai, 400008 India; 2 Department of Mathematics and Sciences, College of Arts and Applied Sciences, Dhofar University, PC 211, Salalah, Sultanate of Oman; 3 Department of Chemistry, University of Mumbai, Vidyanagari, Santacruz (East), Mumbai, 400098 India; 4 College of Engineering, Dhofar University, PC 211, Salalah, Sultanate of Oman; 5 US Army Research Laboratory, Weapons and Materials Research Laboratory, FCDD-RLW-, Aberdeen Proving Ground, Maryland, 21005-5069 USA; 6 Faculty of Chemical and Materials Engineering, Shahrood University of Technology, Shahrood, Iran; 7 School of Mathematics and Physics, College of Science, University of Lincoln, Lincoln, LN6 7TS UK

Retraction of: *Scientific Reports *10.1038/s41598-021-03495-2, published online 07 January 2022

The Editors have retracted this Article.

After publication, concerns were raised regarding the veracity of the following data presented in this work,

- the X-ray diffraction spectra shown in Figure 1;
- the Raman spectra shown in Figure 5(2).

The Authors provided raw data on request from the Editors. However, the Editors found that the original XRD spectra are different from those presented in the Article, and the metadata for the Raman spectra was insufficient to confirm veracity of this data. The Editors therefore no longer have confidence in the research presented in this work.

Riyaz Ahmad Dar, Ashwini Kumar Srivastava, Israr Ul Hassan, and Waqar Ahmed disagree to this retraction. Mashallah Rezakazemi has not stated whether he agrees or disagrees to this retraction. Gowhar Ahmad Naikoo and Ahamad M. H. Shaikh did not reply to correspondence from the Editors. The Editors were unable to contact Shashi P Karna and Lily Giri.

